# Thermography and rasterstereography as a combined infrared method to assess the posture of healthy individuals

**DOI:** 10.1038/s41598-023-31491-1

**Published:** 2023-03-14

**Authors:** Federico Roggio, Luca Petrigna, Bruno Trovato, Marta Zanghì, Martina Sortino, Ermanno Vitale, Lucia Rapisarda, Gianluca Testa, Vito Pavone, Piero Pavone, Michele Vecchio, Giuseppe Musumeci

**Affiliations:** 1 Department of Biomedical and Biotechnological Sciences, Section of Anatomy, Histology and Movement Science, School of Medicine, University of Catania, Via S. Sofia 97, 95123 Catania, Italy; 2 Sport and Exercise Sciences Research Unit, Department of Psychology, Educational Science and Human Movement, University of Palermo, Via Giovanni Pascoli 6, 90144 Palermo, Italy; 3 Occupational Medicine, Department of Clinical and Experimental Medicine, University of Catania, Via Santa Sofia, 87-95123 Catania, Italy; 4 Spine Unit, Cannizzaro Hospital, 95123 Catania, Italy; 5 Department of General Surgery and Medical Surgical Specialties, Section of Orthopedics and Traumatology, AOU. Policlinico-Vittorio Emanuele, University of Catania, Via Santa Sofia 78, 95123 Catania, Italy; 6 Department of Clinical and Experimental Medicine, Pediatric Clinic, University Hospital A.U.O. “Policlinico-Vittorio Emanuele” of Catania, Catania, Italy; 7 Department of Biomedical and Biotechnological Sciences, Section of Pharmacology, University of Catania Rehabilitation Unit, “AOU Policlinico G. Rodolico”, Catania, Italy; 8 Research Center on Motor Activities (CRAM), University of Catania, Via S. Sofia 97, 95123 Catania, Italy; 9 Department of Biology, Sbarro Institute for Cancer Research and Molecular Medicine, College of Science and Technology, Temple University, Philadelphia, PA 19122 USA

**Keywords:** Quality of life, Musculoskeletal system

## Abstract

The demand for noninvasive methods to assess postural defections is increasing because back alterations are more common among the healthy population. We propose a combined infrared method of rasterstereography and thermography to assess the back without harmful effects. This study aims to provide reference data on rasterstereography and thermography to evaluate the back of a healthy population and to further study the correlation between these two methods. This cross-sectional research involved 175 healthy individuals (85 males and 90 females) aged 22 to 35 years. There is a large Cohen’s d effect size in the cervical depth (males = 43.77 ± 10.96 mm vs. females = 34.29 ± 7.04 mm, d = 1.03), and in the lumbar lordosis angle (males = 37.69 ± 8.89° vs. females = 46.49 ± 8.25°, d = − 1.03). The back temperature was different for gender in the cervical area (males = 33.83 ± 0.63 °C vs. females = 34.26 ± 0.84 °C, d = − 0.58) and dorsal area (males = 33.13 ± 0.71 °C vs. females = 33.59 ± 0.97 °C, d = − 0.55). Furthermore, in the female group there was a moderate correlation of lumbar temperature with lumbar lordosis angle (r = − 0.50) and dorsal temperature with shoulders torsion (r = 0.43). Males showed a moderate correlation for vertebral surface rotation RMS with cervical (r = − 0.46), dorsal (r = − 0.60), and lumbar (r = − 0.50) areas and cervical temperature with shoulders obliquity (r = 0.58). These results highlight a possible correlation between rasterstereography and thermography, which may elucidate the underlying mechanics of spinal alterations and thermal muscle response. Our findings may represent reference data for other studies using noninvasive methods to assess postural alterations.

## Introduction

In recent decades, functional assessment of the trunk has increased for both clinical and biomechanical research due to financial and clinical issues. Health institutions demand increasingly reliable and reproducible methods to evaluate a large number of people without harmful effects. According to the Global Burden of Diseases report, musculoskeletal alterations are a leading causes of years lived with disability among young adults^1^. Noninvasive screening methods can detect a specific alteration before the individual experiences discomfort or pain. Rasterstereography is a spreading method that uses light detection and ranging technology (LiDAR) to estimate physiologic or pathological posture. Due to its excellent intra- and interday reliability^2^, it can be considered as a first-level approach when dealing with a large scale of users.

**Figure 1:**
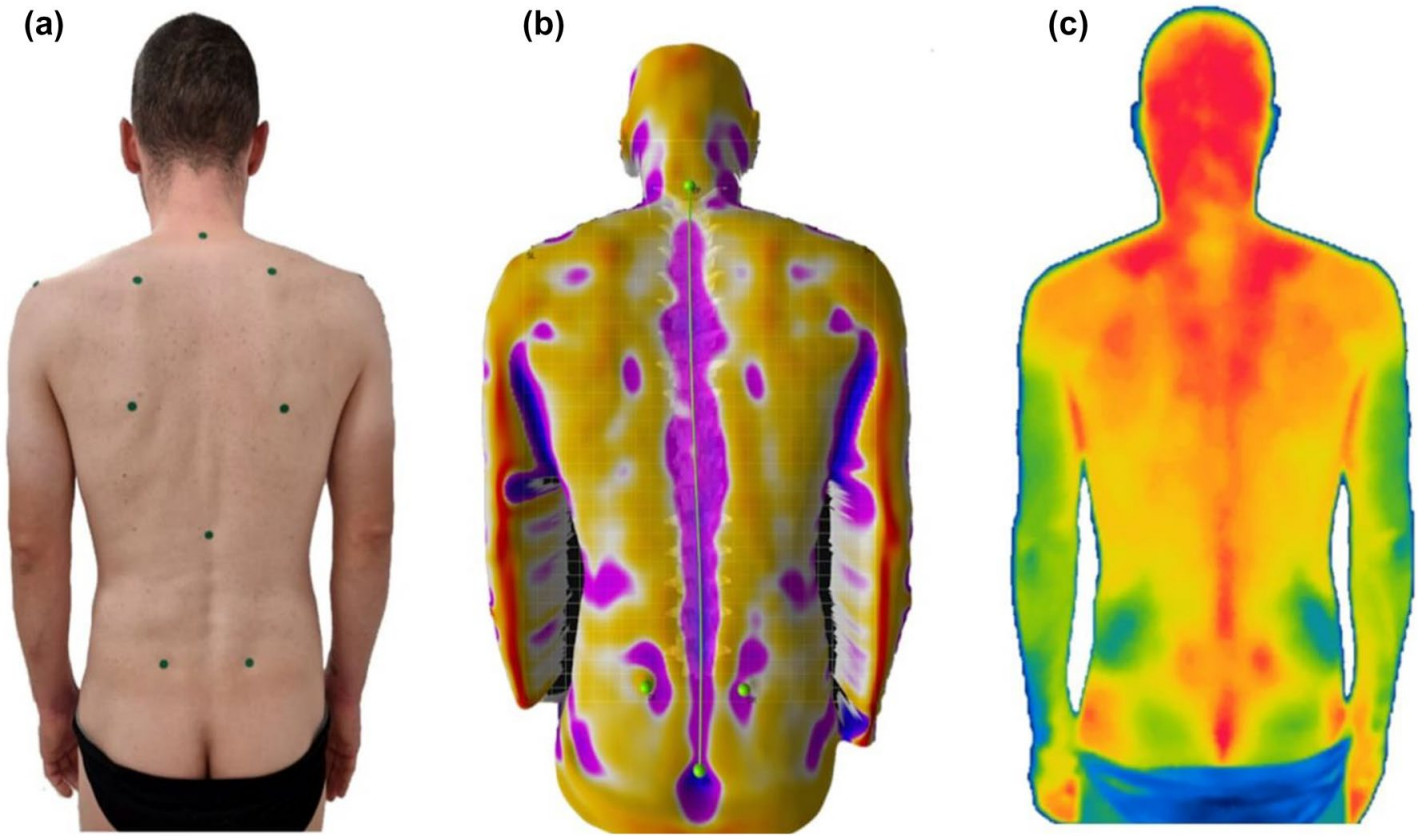
Combined infrared method representing a normal photo of the individual (**a**), a rasterstereographic representation (**b**) deriving from Spine 3D system, and infrared thermography (**c**) obtained from Thermal Studio Pro version number: 1.9.38.0. Image edited with Microsoft Power Point 365, license provided by the University of Catania.

Infrared thermography (IRT) is a noninvasive method valid to investigate the physiological response of the body to different stimuli, e.g. physical activity^3^, rheumatic diseases^4^, and metabolic alterations^5^. Body temperature alteration is a natural indicator of compromised underlying conditions^6^ and muscle demand^7^; IRT is an auxiliary method that supports the diagnosis process by discriminating altered skin temperature and, therefore, physiological processes. We believe that a combined infrared method (CIM) formed by a 3D camera to analyze human movement and thermography to assess thermal symmetry may represent an objective method to analyze the musculoskeletal system, Fig. 1.

In the present study, we employed a CIM to evaluate the back surface of healthy individuals without postural deformities to provide reference data to the research community. Furthermore, we analyzed the correlation between these two noninvasive infrared systems.

## Results

The characteristics of the participants, expressed as mean and standard deviation for body height, body weight, and body mass index (BMI), are presented in Table 1.

**Table 1:** Anthropometric measures of the sample.

	Males	Females	t-test
	Mean ± SD	Mean ± SD	
Age (years)	28.8 ± 6.21	29.6 ± 7.50	< 0.001
Height (cm)	177.37 ± 7.16	163.48 ± 7.20	
Weight (kg)	70.40 ± 7.82	56.19 ± 5.82	
BMI	22.34 ± 1.55	21.01 ± 1.44	

t-test according to Student t-test.

### Rasterstereography

**Figure 2:**
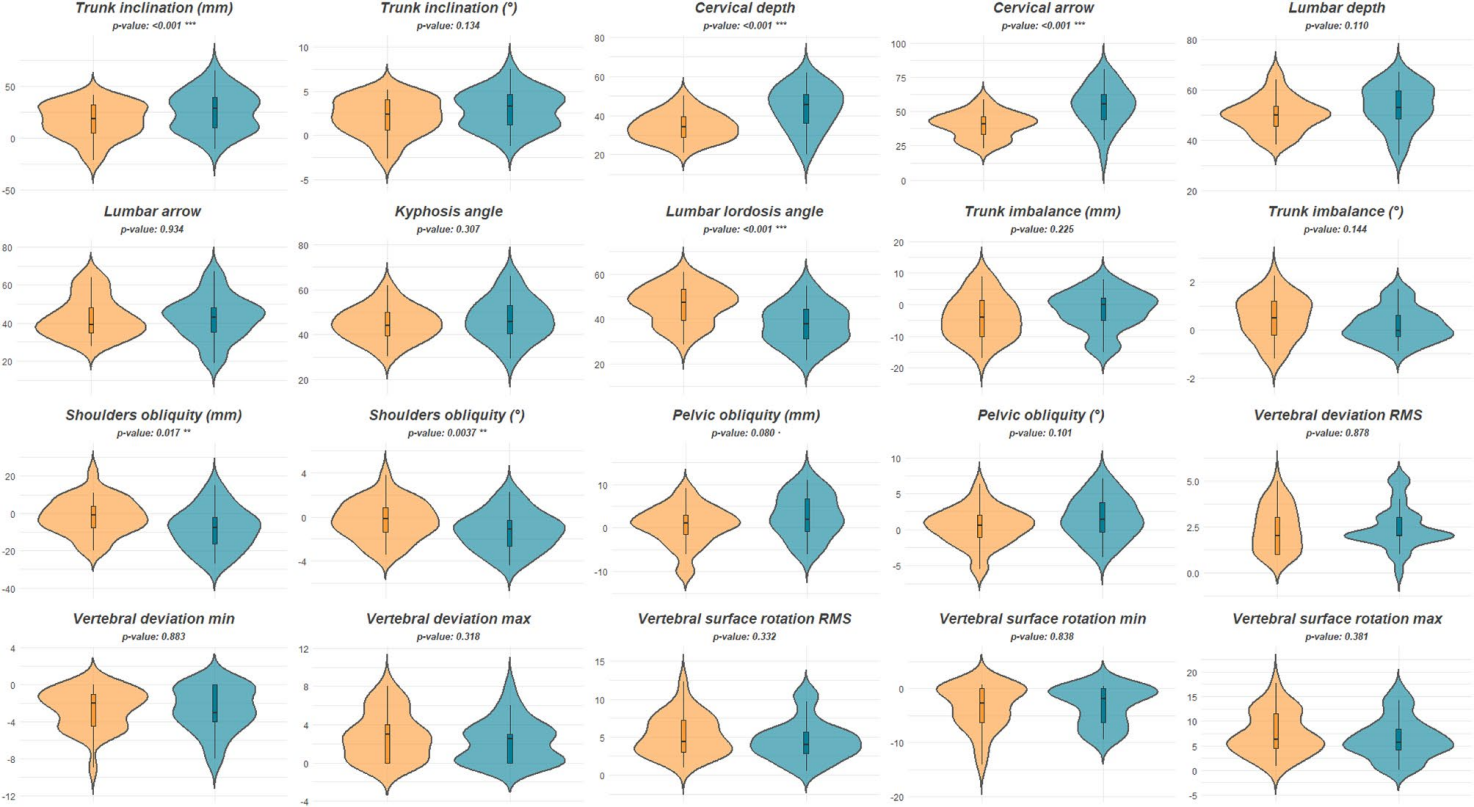
Violin plots for the sagittal (first two lines) and coronal (last three lines) parameters. Orange represents the female group; blue represents the male group. Each violin plot shows inside the boxplot and the correspondent p-value, · p < 0.1, * p < 0.05, ** p < 0.01, *** p < 0.001.

The surface topography results of both the sagittal and coronal planes are reported in Table 2 and Fig. 2 divided by gender; we discuss the results with a p-value < 0.05. On the sagittal plane, the male group shows a higher trunk inclination (31.38 ± 18.90 mm) compared to the female group (20.74 ± 19.95 mm) with a medium effect size (d = 0.55). There is a significant difference in cervical depth between males (43.67 ± 9.99 mm) and females (31.74 ± 7.76 mm) with a large effect size (d = 1.33). This trend is also respected for the cervical arrow, where males (56.97 ± 14.38 mm) have a higher value compared to females (38.73 ± 9.67 mm) with a large effect size (d = 1.49). Finally, the lumbar angle presents a lower value for males (36.39 ± 8.70°) compared to females (47.56 ± 8.47°) with a larger effect value (d = − 1.30). On the coronal plane, we observed only a meaningful difference in shoulders obliquity between males (− 7.23 ± 10.16 mm) and females (− 2.91 ± 9.93 mm) with a medium effect size (d = − 0.43). Finally, we also considered shoulders and pelvic torsion in the transverse plane. The results of the shoulders torsion are 0.34 ± 2.05° for males and 0.07 ± 2.32° (p = 0.634) for females with a small effect size (d = 0.12); the pelvic torsion results are − 1.22 ± 2.97° for males and − 1.81 ± 2.44° for females (p = 0.405) with a small effect size (d = 0.22).

**Table 2:** Rasterstereographic measures of the sagittal and coronal plane.

	Males	Females	Sig.^+^	Effect size (d) ^++^
	Mean ± SD	Mean ± SD		
Sagittal plane				
Trunk length	499.33 ± 29.79	444.94 ± 23.66	< 0.001***	**2.02**
Trunk inclination (mm)	25.53 ± 19.13	16.23 ± 16.64	0.047**	***0.52***
Trunk inclination (°)	2.91 ± 2.14	2.09 ± 2.11	0.134	0.40
Cervical depth	43.77 ± 10.96	34.29 ± 7.04	< 0.001***	**1.03**
Cervical arrow	54.37 ± 15.16	40.32 ± 9.26	< 0.001***	**1.11**
Lumbar depth	53.37 ± 8.65	50.10 ± 7.01	0.110	0.42
Lumbar arrow	42.77 ± 11.62	43.01 ± 10.55	0.934	− 0.02
Kyphosis angle	47.09 ± 9.33	44.85 ± 7.48	0.307	0.26
Lumbar lordosis angle	37.69 ± 8.89	46.49 ± 8.25	< 0.001***	**− 1.03**
Coronal plane				
Trunk imbalance (mm)	− 1.9 ± 6.14	− 3.94 ± 6.83	0.225	0.31
Trunk imbalance (°)	0.21 ± 0.69	0.51 ± 0.89	0.144	− 0.38
Shoulders obliquity (mm)	− 8.23 ± 11.11	− 1.68 ± 9.71	0.017**	***− 0.63***
Shoulders obliquity (°)	− 1.26 ± 1.73	− 0.31 ± 1.75	0.037**	***− 0.55***
Pelvic obliquity (mm)	2.73 ± 4.93	0.58 ± 4.51	0.080**·**	***0.51***
Pelvic obliquity (°)	1.64 ± 2.89	0.46 ± 2.62	0.101	0.43
Vertebral deviation RMS	2.47 ± 1.2	2.42 ± 1.20	0.878	0.04
Vertebral deviation min	− 2.67 ± 2.41	− 2.58 ± 2.16	0.883	− 0.04
Vertebral deviation max	2.2 ± 2.22	2.81 ± 2.48	0.318	− 0.26
Surface rotation RMS	4.57 ± 2.65	5.23 ± 2.66	0.332	− 0.25
Surface rotation min	− 3.22 ± 3.46	− 3.42 ± 4.08	0.838	0.05
Surface rotation max	6.49 ± ± 4.23	7.45 ± 4.31	0.381	− 0.22

^+^according to t-test for normal data and Mann–Whitney U for non-normal data **·** p < 0.1, * p < 0.05, ** p < 0.01, *** p < 0.001.

^++^Cohen’s value, bold numbers indicate a large effect size between groups (d > 0.80). Bold and italic numbers indicate a medium effect size between groups (d > 0.50).

### Infrared thermography

**Figure 3:**
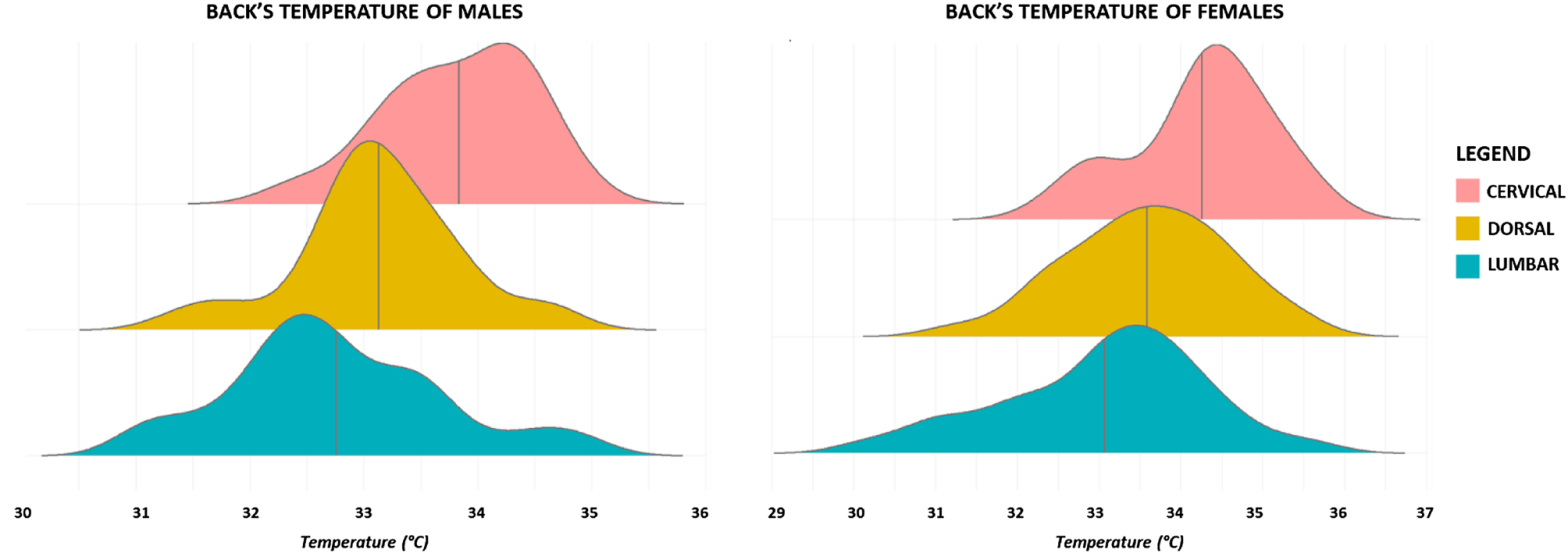
Ridge plots of temperature distribution for males and females. The vertical line in each plot represents the mean temperature.

Males have a lower cervical temperature (33.83 ± 0.63 °C) compared to females (34.26 ± 0.84 °C) with p = 0.029 and a medium effect size (d = − 0.58). Furthermore, the dorsal temperature of males (33.13 ± 0.71 °C) is lower compared to females (33.59 ± 0.97 °C) with p = 0.035 and medium effect size (d = − 0.55). The lumbar temperature of males (32.76 ± 0.94 °C) and females (33.06 ± 1.23 °C) does not differ between the groups, with p = 0.273 and a low effect size (d = − 0.27). However, the data distribution is different between the groups, Fig. 3.

#### Correlation between rasterstereography and infrared thermography

IRT measures have been correlated with rasterstereography measures to observe if a higher or lower skin temperature may reflect a correlation with back topography. The male group showed a negative correlation between lumbar temperature and trunk imbalance (°) (r = − 0.42, p = 0.032); vertebral surface rotation RMS with cervical (r = − 0.46, p = 0.010), dorsal (r = − 0.60, p < 0.001) and lumbar (r = − 0.50, p = 0.007) temperatures; vertebral surface rotation max with cervical (r = − 0.45, p = 0.013), dorsal (r = − 0.56, p = 0.001) and lumbar (r = − 0.38, p = 0.043) temperatures. Meanwhile, they present a positive correlation between shoulders obliquity with cervical temperature (r = 0.58, p < 0.001) and with dorsal temperature (r = 0.45, p = 0.020).

The female group showed a negative correlation between lumbar temperature with lumbar lordosis angle (r = − 0.50, p = 0.004). Instead, dorsal temperature is positively correlated with trunk imbalance (°) (r = 0.42, p = 0.022); lumbar temperature with trunk imbalance (°) (r = 0.43 p = 0.016); dorsal temperature with shoulders torsion (r = 0.43, p = 0.014).

## Discussion

This study aimed to present reference data on physiological posture standards of healthy individuals without spinal deformities using a CIM. Rasterstereography evaluated the back topography; the IRT measured the thermal emissivity of the back to assess muscle activity. These two methods have spread in recent years thanks to the ease of use and objective measures that can support the clinical practice of analyzing the spine and detecting underlying conditions not yet visible to the human eye, as claimed by two systematic reviews^8,9^. The demand for rasterstereography as a noninvasive method is increasing to reduce the burden of the healthcare system and reduce follow-up radiological measurements^10^. Similarly, IRT is providing valuable results in monitoring the body's response to external stimuli such as cryotherapy^11^, whole-body vibration^12^, and strength training^13^.

Establishing a thermal profile has been one of the main topics since IRT was adopted for human diagnostic purposes. In the late 1980s, Uematsu et al.^14,15^ tried to quantify the thermal symmetry of healthy individuals by studying the differences between both sides of the body. Even if these initial results were promising, the limitations of the tools of that period stalled its progression. Nowadays, different authors, through modern IR cameras, have attempted to classify different body areas among young adults. Chudecka and Lubkowska^16^ analyzed the IRT of 100 males and 100 females (aged 20–23), finding that only the chest area had a higher temperature in females, while the other areas were warmer in males. The mean temperature of the upper back of males (33.92 ± 0.19 °C) is similar to our results for the cervical area (33.83 ± 0.63 °C). Marins et al.^17^, aiming to present normative data of healthy Brazilian adults (mean age 21.6 ± 2.2), found a significant gender difference in the thigh region while there was no difference in the hands, leg, abdomen, and lower back, as our results. In another study, Marins et al.^18^ accomplished the IRT in the early morning (7 a.m.) and late evening (7 p.m.) of military males and females. They found a gender difference in the morning thermograms, while no differences relative to gender were present in the evening collections.

We compared the results of the rasterstereography with four similar studies^19–22^ evaluating the back of healthy individuals, as reported in Table 3. Our findings showed a general difference based on gender for almost all parameters of the rasterstereography of the sagittal plane; meanwhile, only three parameters of the coronal plane differed by gender. We found a great difference in cervical measures; males show an increased depth of the cervical area, a trend also respected for the lumbar depth. However, the lumbar lordosis angle appears to be greater for females, similar to the results of Michalik et al.^21^. Meanwhile, the lumbar lordosis angle of the males is similar to the results of Degenhardt et al.^19,20^. Although the trunk inclination of the females is similar to the results of Michalik et al.^21^, the values of the males are similar only to the studies of Degenhardt et al.^11,12^. For the parameters of the coronal plane, the only similarities are in the pelvic obliquity of our females with the findings of Michalik et al.^21^. According to our findings, there is a significant difference in trunk length between males and females, which may explain some of the differences found in other parameters. This difference is likely due to the biological differences between males and females, as well as differences in their activities or occupations. The morphological characteristics of the body play an important role in determining its stability and posture^23^. We suggest that the differences in trunk inclination and sagittal curvatures, such as cervical depth and lumbar lordosis angle differences, are a response to the body's evolution and environment^24^. The increase in trunk inclination is essential for maintaining the center of gravity within the base of support, and men are usually found to have a larger sway amplitude compared to women^23^. Previous studies^25,26^ have also observed gender differences in anthropometry, vertebral geometry, and strength of the neck and shoulder area, which may contribute to the observed differences in posture. For example, women generally have smaller vertebrae and weaker muscles compared to men. Our findings are consistent with these observations, as we found similar gender differences in both rasterstereography and IRT measurements.

**Table 3:** Parameters comparison of mean ± SD with other studies.

	Males	Females	Degenhardt et al.^19^	Degenhardt et al.^20^	Michalik et al.^21^ (Males)	Michalik et al.^21^ (Females)	Wolf et al.^22^
Sagittal plane							
Trunk length	499.33 ± 29.79	444.94 ± 23.66	463.35 ± 33.38	466 ± 33.3	492.82 ± 28.43	452.38 ± 26.72	n.a
Trunk inclination (mm)	25.53 ± 19.13	16.23 ± 16.64	25.49 ± 18.32	26.23 ± 17.66	n.a	n.a	25.7 ± 16.9
Trunk inclination (°)	2.91 ± 2.14	2.09 ± 2.11	3.09 ± 2.25	3.17 ± 2.18	1.89 ± 1.88	2.12 ± 2.40	3.2 ± 2.1
Cervical depth	43.77 ± 10.96	34.29 ± 7.04	n.a	n.a	n.a	n.a	n.a
Cervical arrow	54.37 ± 15.16	40.32 ± 9.26	71.08 ± 19.67	74.42 ± 16.49	n.a	n.a	n.a
Lumbar depth	53.37 ± 8.65	50.10 ± 7.01	n.a	n.a	n.a	n.a	n.a
Lumbar arrow	42.77 ± 11.62	43.01 ± 10.55	36.62 ± 12.62	37.53 ± 12.18	n.a	n.a	n.a
Kyphosis angle	47.09 ± 9.33	44.85 ± 7.48	47.23 ± 9.35	48.47 ± 8.32	44.58 ± 7.84	44.02 ± 8.64	44.2 ± 7.9
Lumbar lordosis angle	37.69 ± 8.89	46.49 ± 8.25	36.26 ± 8.53	35.42 ± 7.55	28.96 ± 7.67	37.36 ± 28.96	41.5 ± 9.2
Coronal plane							
Trunk imbalance (mm)	− 1.9 ± 6.14	− 3.94 ± 6.83	1.32 ± 7.16	1.29 ± 5.62	n.a	n.a	− 2.6 ± 7.5
Trunk imbalance (°)	0.21 ± 0.69	0.51 ± 0.89	0.16 ± 0.85	0.15 ± 0.66	− 0.08 ± 0.96	− 0.07 ± 0.91	− 0.3 ± 0.9
Shoulders obliquity (mm)	− 8.23 ± 11.11	− 1.68 ± 9.71	n.a	n.a	n.a	n.a	− 7.3 ± 8.9
Shoulders obliquity (°)	− 1.26 ± 1.73	− 0.31 ± 1.75	n.a	n.a	n.a	n.a	− 1.1 ± 1.3
Pelvic obliquity (mm)	2.73 ± 4.93	0.58 ± 4.51	− 0.11 ± 3.39	− 0.12 ± 5.13	n.a	n.a	− 0.2 ± 2.2
Pelvic obliquity (°)	1.64 ± 2.89	0.46 ± 2.62	0.00 ± 5.78	− 0.17 ± 2.92	− 0.32 ± 3.34	− 0.42 ± 2.79	− 0.1 ± 1.1
Vertebral deviation RMS	2.47 ± 1.2	2.42 ± 1.20	5.53 ± 2.92	5.43 ± 2.49	5.07 ± 2.13	5.59 ± 2.32	n.a
Vertebral deviation min	− 2.67 ± 2.41	− 2.58 ± 2.16	− 4.73 ± 4.11	8.04 ± 5.13	n.a	n.a	− 4.4 ± 3.6
Vertebral deviation max	2.2 ± 2.22	2.81 ± 2.48	7.86 ± 5.60	− 4.62 ± 2.92	n.a	n.a	3.2 ± 3.0
Surface rotation RMS	4.57 ± 2.65	5.23 ± 2.66	3.74 ± 1.24	3.78 ± 0.93	3.54 ± 1.56	3.64 ± 1.62	n.a
Surface rotation min	− 3.22 ± 3.46	− 3.42 ± 4.08	− 4.38 ± 2.71	− 4.51 ± 2.40	n.a	n.a	− 3.9 ± 2.8
Surface rotation max	6.49 ± ± 4.23	7.45 ± 4.31	5.97 ± 3.51	5.68 ± 2.79	n.a	n.a	1.7 ± 1.9

*n.a* not available, columns males (n = 85) and females (n = 90) represent our findings, Degenhardt et al.^19^ (n = 30 M/F), Degenhardt et al.^20^ (n = 30 M/F), Michalik et al.^21^ males = 65, females = 56), Wolf et al.^22^ (n = 100 females).

Finally, we correlated the rasterstereography parameters with the IRT. Even if with moderate strength, the correlations reported are all statistically significant, meaning that they are unlikely to have occurred by chance. In the female group, when the lumbar lordosis angle increases, the lumbar temperature decreases. Studies have shown that the lumbar lordosis angle is genetically different between males and females, with females having a greater angle^27,28^. In our study, we observed a reduction in anterior imbalance, which was balanced by an increase in the lumbar lordosis angle. Since this is an anatomical aspect and not an acquired condition, it does not involve muscle activity, which results in lower metabolic activity in the underlying muscles, and thus a possible explanation for the negative correlation. The dorsal temperature was positively correlated with shoulder torsion. We suggest that wearing uncomfortable bras could lead to a constant postural defect, which may cause torsion of the shoulders. As Chen et al.^29^ observed, different types of bras can restrict shoulder motion and cause discomfort. Therefore, the increased temperature may be related to a higher demand of the body to support the breast. Finally, also lateral trunk imbalance was positively correlated with lumbar and dorsal temperatures, suggesting that as the degree of trunk imbalance increases, the temperature in the lumbar and dorsal regions tends to increase. This may be due to an increase in muscle activity in the lumbar and dorsal regions to compensate for trunk imbalances, leading to increased metabolic activity and subsequent elevation of skin temperature in these regions. This phenomenon may also be related to the previous statement.

In our male group, shoulder obliquity was moderately correlated with cervical and dorsal temperature. Since they practice gym activities, we hypothesized that these positive correlations, i.e., as the shoulder obliquity increases, the temperature increases, could be explained by the higher muscular demand of the shoulders area. As males tend to work out their upper limbs more than females^30^, this may contribute to the higher temperature in the shoulder region. Then, we observed a moderate negative correlation between cervical, dorsal and lumbar temperatures with both vertebral surface rotation RMS and maximum rotation. In scoliosis, the concave side is the side toward which the vertebrae rotate, and, as asserted by Kwok et al.^31^, the concave side of scoliosis has a lower temperature. Our results highlight a trend, when the vertebrae rotation increases, the temperature decreases. However, it is important to note that these findings were observed only in the male group and should be interpreted with caution. Finally, we found that the negative correlation between lumbar temperature and trunk imbalance may be related to muscle imbalances caused by gym activities^32^. Although as a stand-alone consideration may be meaningless, when we also consider the valuable correlation between vertebral surface rotation and skin temperatures, it suggests that males may be at risk of spinal misalignment. Thus, a decrease in skin temperature may be associated with an increase in spinal deformities or muscle imbalances.

These findings suggest that different mechanisms may influence the relationship between skin temperature and back topography in males and females, potentially, potentially due to differences in muscle activation and blood flow regulation between the genders. However, more research is needed to fully understand the underlying mechanisms driving these correlations.

Currently, both rasterstereography and IRT are being studied in the evaluation and progression of scoliosis, even if there are still some concerns. The former is not sufficiently accurate to diagnose scoliosis, but as observed by different authors, it is making considerable progress in characterizing the typical signs of scoliosis, such as vertebral rotation^33^, shoulder imbalance^34^, and monitoring the progression of scoliosis^35^. The latter is yielding promising results for scoliosis evaluation^36^, highlighting its feasibility for school scoliosis screening, a field where preventive care is required^37^.

Aware of the impossibility of considering rasterstereography as a substitute for x-rays in the diagnosis of spinal pathologies^25^, we support its strength as a screening tool, as reported by Rusnak et al. in the early identification of spinal deformities in 311 children^38^. Likewise, we support IRT as a complementary method for screening and preventing muscle injuries^39^ and inflammatory processes^40^. Therefore, we believe that reference data from both screening techniques can support orthopaedic, rehabilitation, and clinical research toward a better distinction of red flags of spine deformities.

This study has some limitations. First, we observed a group of healthy adults with similar anthropometrics under 35 years of age, so the findings should be carefully interpreted when comparing them with pathological patients or old adults. Second, although the participants did not present any detectable posture alteration, it was not checked with diagnostic tools (x-rays or MRI), so there may be some minor posture alterations. Third, we did not analyze fat tissue, so even if individuals with BMI > 25 were not considered, we could not be sure that the temperature was the same for all the participants. Further studies are required to investigate the thermal changes associated with fat tissue percentage by conducting bioelectrical impedance analysis and considering different age ranges, e.g., adolescents and older adults.

We believe that this pioneering technique, a combination of infrared methods, will aid in elucidating the characteristics of posture alterations and muscle activity with a non-invasive, easy, and reliable method. Future studies could use it to study musculoskeletal pathologies and gait analysis and discover correlations between IRT and kinematics and kinetics.

## Conclusions

A CIM composed of rasterstereography and thermography has been adopted to study the postural assessment of the back classified by gender. Males commonly present a higher trunk inclination, shoulder obliquity, cervical, and lumbar depth. Although the kyphosis angle is the same for both sexes, females present an increased lumbar lordosis angle. Females have a significantly higher temperature in the cervical and dorsal areas of the back compared to males, while the lumbar temperature is also higher in females but not statistically significant. The correlation between these two methods requires further investigation as it may help to better understand the complex mechanism of spine alterations and muscle activity asymmetry. This study is a significant contribution to knowledge on back topography and may be a reference for other researchers interested in using a CIM to evaluate postural alterations.

## Materials and methods

This cross-sectional study involved 175 healthy individuals (85 males and 90 females) aged 22 to 35 and analyzed the back surface with rasterstereography and thermography. Participants were recruited voluntarily at the Research Center on Motor Activities (CRAM), University of Catania. We considered the age limit of 35 years to avoid confounding elements due to the incidence of age-related musculoskeletal disorders^1^ or specific work conditions that can bias the data. Participants completed a questionnaire to collect general information about pathologies, allergies, medication use, recent surgery, regular menstrual cycle, sports played, and dominant limb. According to this information, the exclusion criteria were musculoskeletal disorders, history of scoliosis or spine alterations, acute back pain during the previous four months, recent surgery, altered menstrual cycle, BMI < 18.5 or > 25. The study was approved by the Research Center on Motor Activities (CRAM) Scientific Committee (Protocol n.: CRAM-020-2021, 20 December 2021), in accordance with the Declaration of Helsinki. All participants provided their informed consent prior to participating.

### Data collection

**Figure 4:**
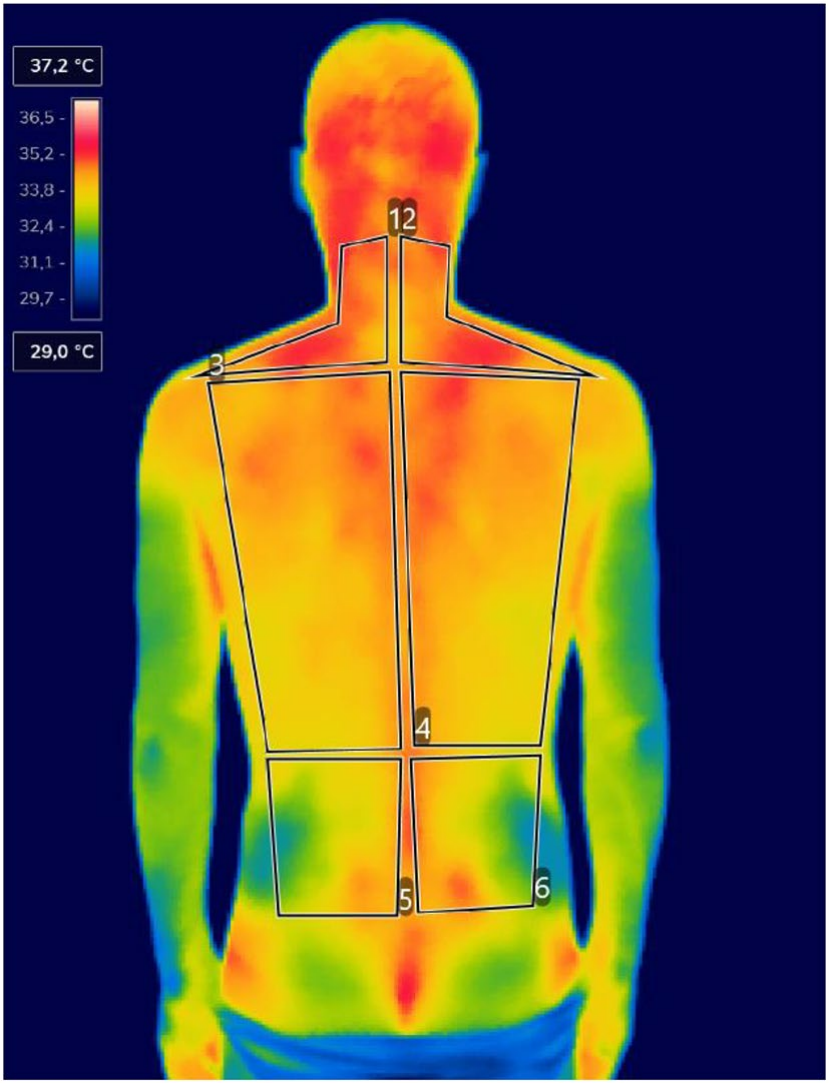
Representation of IRT acquisition and polygon division of each back’s area. Numbers 1 and 2 represent the cervical area, numbers 3 and 4 the dorsal area, numbers 5 and 6 the lumbar area. Thermogram edited with the software Thermal Studio PRO, version number: 1.9.38.0.

A LiDAR technology was used to assess the rasterstereography. The Spine 3D (Sensormedica, Rome, Italy) is a noninvasive 3D system that analyzes the spine in the three planes: sagittal, coronal, and transverse, with an excellent intra-day and inter-day reliability in almost all parameters^41^. A 3D camera embedded in the system evaluates the back with the time of flight method, with a resolution of 1920 × 1080 pixels and a frame rate of acquisition of 30 fps. A detailed explanation of all the parameters collected is reported in Table 4. The IR acquisitions were carried out according to the TISEM checklist^42^ to ensure the quality of thermal images and reduce bias. IR images were taken with FLIR E54 camera (Wilsonville, OR, USA) camera with a detector resolution of 320 × 240 pixels and thermal sensitivity < 0.04 °C. The camera was placed on a tripod, positioned 1.5 m away from the individual in a room with a temperature of 24 ± 2 °C and humidity of 50%; emissivity level was set at 0.98. Infrared thermography detects the radiance of a body; then, the algorithms present in these cameras convert the radiance into temperature values, thus providing the expression of the temperature of the body surface^43^. The participants were asked to rest for 15 min before the IR imaging was taken in order to allow for acclimatization. For both acquisitions, participants were instructed to stand upright with their back to the camera, arms by their side, without upper clothes, and buttocks slightly uncovered. For the rasterstereography acquisition, participants were instructed to place the heels on a line 110 cm from the camera, looking straight ahead. For the IRT acquisition, participants were instructed to stand upright with the arms slightly away from the trunk. The IRT camera was placed 150 cm away from the participant. Each of the thermograms was analyzed using FLIR Thermal Studio PRO software, version number: 1.9.38.0. The regions of interest were the left and right sides of the cervical, dorsal, and lumbar area, Fig. 4. We strictly followed the suggestion from the practical guide of Ammer and Ring^44^ to avoid possible bias in the study. Thermograms whose difference between the left and right side was > 0.3 °C were excluded^45^.

**Table 4:** Description of rasterstereography parameters.

Sagittal plane	
Trunk length (mm)	The distance between VP and DM
Trunk inclination (mm)	The distance between two vertical lines passing for VP and DM
Trunk inclination (°)	The angle between the plumb line passing for VP and a vertical line connecting VP-DM
Cervical depth	The horizontal distance between the cervical apex and a tangent passing for KA
Cervical arrow	The horizontal distance between the cervical apex and a perpendicular line passing for KA
Lumbar depth	The horizontal distance between the lumbar apex and a tangent passing for KA
Lumbar arrow	The horizontal distance between the lumbar apex and a perpendicular line passing for KA
Kyphosis angle	The angle formed between the two surface tangent lines of the ICT and ITL
Lumbar lordosis angle	The angle formed between the two surface tangent lines of the ITL and ILS
Coronal plane	
Trunk imbalance (mm)	The lateral distance between two lines passing for VP and DM
Trunk imbalance (°)	The angle between the plumb line passing for VP and a vertical line connecting VP-DM
Shoulders obliquity (mm)	The distance between two horizontal lines passing for SR and SL
Shoulders obliquity (°)	The angle between a horizontal line passing for SR and SL and a horizontal line perpendicular to the gravity line
Pelvic obliquity (mm)	The distance between two horizontal lines passing for DR and DL
Pelvic obliquity (°)	The angle between a horizontal line passing for DR and DL and a horizontal line perpendicular to the gravity line
Vertebral deviation RMS	The RMS deviation of the midline of the spine from the direct connection VP-DM line
Vertebral deviation min	The maximum deviation to the left of the midline of the spine from the VP-DM line
Vertebral deviation max	The maximum deviation to the right of the midline of the spine from the VP-DM line
Surface rotation RMS	The RMS rotation in the axial plane of a spinous process when compared to the neutral pelvis
Surface rotation min	The maximum rotation to the left in the axial plane of a spinous process when compared to the neutral pelvis
Surface rotation max	The maximum rotation to the right in the axial plane of a spinous process when compared to the neutral pelvis
Transversal plane	
Shoulder torsion	The rotation in the transversal plane of the SR relative to a reference coronal plane perpendicular to the camera-projection axis
Pelvic torsion	The rotation in the transversal plane of the DR relative to a reference coronal plane perpendicular to the camera-projection axis

*VP* vertebra prominent, *DM* dimple midpoint, *KA* kyphotic apex, *ICT* inflection point between cervical and thoracic spine, *ITL* inflection point between thoracic and lumbar spine, *ILS* inflection point between lumbar spine and sacrum, *SR* shoulder right, *SL* shoulder left, *DR* dimple right, *DL* dimple left, *RMS* root mean square.

### Statistical analysis

The data analysis was conducted using R Project for Statistical Computing (Vienna, Austria). The Shapiro–Wilk test verified the normality distribution, the Student t-test and Mann–Whitney U were used to determine whether any significant difference was present between males and females for rasterstereography and IRT imaging. Cohen's effect size (d) identified significant differences between the groups. Pearson correlation coefficients (r) were calculated to estimate correlations between rasterstereography and the surface temperature of the selected regions of interest.

### Informed consent

Informed consent was obtained from all subjects involved in the study.

### Institutional review board statement

The study was conducted in accordance with the Declaration of Helsinki and approved by the Scientific Committee of the Research Center on Motor Activities (CRAM) (Protocol n.: CRAM-020-2021, 20 December 2021).

## Data Availability

The datasets used and/or analyzed during the current study available from the corresponding author on reasonable request.
